# Biomarkers of inflammation, coagulation and fibrinolysis predict mortality in acute lung injury

**DOI:** 10.1186/cc6846

**Published:** 2008-03-21

**Authors:** Dana McClintock, Hanjing Zhuo, Nancy Wickersham, Michael A Matthay, Lorraine B Ware

**Affiliations:** 1 Cardiovascular Research Institute, 505 Parnassus Avenue, University of California, San Francisco, San Francisco, CA 94143, USA; 2 Division of Allergy, Pulmonary and Critical Care Medicine, Department of Medicine, Vanderbilt University, T1218 MCN, 1161 21^st ^Avenue S, Nashville, TN 37232-2650 USA

## Abstract

**Background:**

Acute lung injury (ALI) is a major cause of acute respiratory failure with high mortality despite lung-protective ventilation. Prior work has shown disordered inflammation and coagulation in ALI, with strong correlations between biomarker abnormalities and worse clinical outcomes. We measured plasma markers of inflammation, coagulation and fibrinolysis simultaneously to assess whether these markers remain predictive in the era of lung-protective ventilation.

**Methods:**

Plasma samples and ventilator data were prospectively collected from 50 patients with early ALI. Plasma biomarkers of inflammation (IL-6, IL-8, intercellular adhesion molecule 1), of coagulation (thrombomodulin, protein C) and of fibrinolysis (plasminogen activator inhibitor 1) were measured by ELISA. Biomarker levels were compared between survivors (n = 29) and non-survivors (n = 21) using Mann–Whitney analysis.

**Results:**

The tidal volume for the study group was 6.6 ± 1.1 ml/kg predicted body weight and the plateau pressure was 25 ± 7 cmH_2_O (mean ± standard deviation), consistent with lung-protective ventilation. All markers except IL-6 were significantly different between survivors and nonsurvivors. Nonsurvivors had more abnormal values. Three biomarkers – IL-8, intercellular adhesion molecule 1 and protein C – remained significantly different by multivariate analysis that included age, gender, Simplified Acute Physiology Score II and all biomarkers that were significant on bivariate analysis. Higher levels of IL-8 and intercellular adhesion molecule 1 were independently predictive of worse outcomes (odds ratio = 2.0 and 5.8, respectively; *P *= 0.04 for both). Lower levels of protein C were independently associated with an increased risk of death (odds ratio = 0.5), a result that nearly reached statistical significance (*P *= 0.06).

**Conclusion:**

Despite lung-protective ventilation, abnormalities in plasma levels of markers of inflammation, coagulation and fibrinolysis predict mortality in ALI patients, indicating more severe activation of these biologic pathways in nonsurvivors.

## Introduction

Acute lung injury (ALI) and acute respiratory distress syndrome (ARDS) are common causes of acute respiratory failure with a high mortality rate despite decades of research into these conditions [1]. Many studies have implicated activation of inflammation and derangement of the coagulation and fibrinolytic pathways in patients with ALI/ARDS. A number of biomarkers of inflammation are associated with poor clinical outcomes in patients with ALI/ARDS, including intercellular adhesion molecule 1 (ICAM-1), IL-6 and IL-8 [2-4]. In patients with ALI/ARDS from a variety of predisposing conditions, higher levels of the proinflammatory cytokines IL-6 and IL-8 predict worse outcomes [5]. Moreover, levels of ICAM-1 (unpublished data), IL-6 and IL-8 [5] and levels of other proinflammatory cytokines [6] are reduced by a low-tidal-volume ventilatory strategy.

In addition to inflammatory markers, markers of dysregulated coagulation and fibrinolysis are predictive of clinical outcomes in patients with ALI/ARDS. Protein C is an endogenous anticoagulant and antiinflammatory protein that is activated by binding to the thrombin–thrombomodulin complex on the endothelium. Lower levels of protein C and higher levels of circulating thrombomodulin are consistent with a procoagulant state [7]. Fibrinolysis, the process of resolving clot formation, is also impaired in patients with ALI/ARDS [8,9]. In a larger multicenter study, higher levels of plasminogen activator inhibitor 1 (PAI-1) and lower levels of protein C in the plasma had a synergistic association with higher mortality in patients with ALI/ARDS [10]. Protein C levels increased in patients treated with a low-tidal-volume ventilatory strategy in the study.

Given the importance of inflammation and coagulation to the pathogenesis of ALI/ARDS and the demonstrated improvement in biomarkers in these pathways in patients treated with lower tidal volumes, we investigated whether biomarkers of a proinflammatory, procoagulant and antifibrinolytic state remain predictive in the era of routine use of low-tidal-volume ventilation for ALI/ARDS. We chose to test multiple markers within the inflammatory and coagulation cascades that have a carefully considered pathogenetic basis in ALI/ARDS. Our hypothesis was that, despite effective institution of a lung-protective ventilatory strategy, derangement in the plasma levels of biomarkers reflecting inflammation and disordered coagulation and fibrinolysis would be associated with increased mortality in a cohort of prospectively collected patients with ALI/ARDS.

## Materials and methods

### Subjects and patient samples

Fifty patients who met the American–European Consensus Conference definition for ALI or ARDS [11] were recruited from both Moffitt-Long University Hospital (33 patients) and San Francisco General Hospital (17 patients) from 2003 to 2006. Patients were recruited for participation within 48 hours of meeting the diagnostic criteria for ALI or ARDS. Informed consent for study participation was obtained from each subject or their designated surrogate. In the case of surrogate consent, follow-up consent was sought from the subject whenever possible. The study was approved by the Committee on Human Research at the University of California San Francisco and was performed in compliance with the mandates of the Helsinki Declaration.

Clinical data, including severity of illness scores and risk factors for the development of ALI/ARDS, were abstracted from the medical record. Ventilator data were also recorded for each subject at the time of collection of the plasma samples. The tidal volume was expressed as the tidal volume per kilogram of predicted body weight [12]. The primary outcome for the present study was in hospital mortality. The University of California San Francisco Committee on Human Research approved the study protocol.

### Plasma biomarker measurements

Plasma samples were collected from each patient at the time of enrollment with the *pre hoc *intent to study biomarkers of inflammation and coagulation. Blood was collected in heparin tubes and centrifuged for 10 minutes at 3,000 × *g*. Plasma supernatant was removed from the spun samples and was frozen at -70°C until the time of analysis. The analyses included markers of inflammation, coagulation and fibrinolysis. Specifically, we measured the inflammatory biomarkers ICAM-1, IL-6 and IL-8 and markers of disordered coagulation and fibrinolysis, including protein C, thrombomodulin and PAI-1. An ELISA was used to measure each biomarker in duplicate: ICAM-1, IL-6 and IL-8 (R&D Systems, Minneapolis, MN, USA); thrombomodulin and PAI-1 (American Diagnostica, Stamford, CT, USA); and Protein C (Helena Laboratories, Beaumont, TX, USA).

### Statistical analysis

All statistical analyses were performed using STATA software (StataCorp, College Station, TX, USA). All analyses compared survivors with nonsurvivors in this group of patients. For baseline demographics and clinical data, we used chi-square analysis for dichotomous predictor variables and used an unpaired *t *test to compare survivors and nonsurvivors.

Biomarker values were not normally distributed. Logarithmic transformation of the biomarker data did not normalize the data as assessed by the Shapiro–Wilk test of normalcy. Hence, bivariate analysis of the association between biomarker values and the outcome of mortality was assessed using nonparametric analysis, specifically Mann–Whitney analysis.

We subsequently performed a logistic regression analysis to assess the contribution of demographic, clinical and biomarker data to the outcome of mortality. We confirmed these findings with a stepwise logistic regression model that included sepsis as a condition predisposing to ALI/ARDS to determine significant independent contributors to mortality in ALI/ARDS patients. Sepsis was included in the model since sepsis alone is recognized to contribute to increased mortality [1] as well as to abnormalities in biomarker levels. Statistical significance for each of these analyses was defined as *P *< 0.05.

## Results

### Demographic, clinical and ventilator parameters

Baseline demographic data and clinical variables are presented in Table 1. The ventilator parameters are presented in Table 2. The ventilator parameters were similar to the ventilator parameters reported for the 6 ml/kg tidal volume group in the ARDS Network trial of lower tidal volume ventilation [12]. The mean time between meeting diagnostic criteria for ALI/ARDS and obtaining the day 1 plasma sample for this patient group was 50 hours.

**Table 1:** Demographic and clinical data

Parameter	Current study population value (n = 50)
Demographics	
Age (years) (mean ± standard deviation)	55 ± 16
Gender (% male)	56
Race/Ethnicity	
Asian (%)	14
African American (%)	16
Hispanic (%)	16
Caucasian (%)	54
Conditions predisposing to acute lung injury	
Sepsis (%)	36
Pneumonia (%)	34
Aspiration of gastric content (%)	16
Transfusion of blood products (%)	4
Other^a ^(%)	10
Hospital mortality (%)	42

^a^Other group included pancreatitis, near drowning, smoke inhalation, drug overdose and post-surgical complication.

**Table 2:** Ventilator settings and physiologic parameters at enrollment

Parameter	Current study population value
Number of patients	50
Tidal volume (per kg predicted body weight)	6.6 ± 1.1
Plateau pressure (cmH_2_O)	25 ± 7
PaO_2_/FIO_2 _ratio	155 ± 72
Positive end expiratory pressure (cmH_2_O)	10 ± 4
Mean airway pressure (cmH_2_O)	17 ± 5

### Comparison of demographic, clinical and ventilator results by survival group

Survivors and nonsurvivors were similar in terms of age, gender and racial distribution. There were more patients with sepsis in the group that did not survive (Table 3). Markers of severity of disease, including the Acute Physiology and Chronic Health Evaluation II score [13] and the Simplified Acute Physiology Score II (SAPS II) [14], were higher in nonsurvivors, although this finding only reached statistical significance for the SAPS II score (*P *= 0.02). There were no differences in the plateau pressure, the quasistatic respiratory compliance, the PaO_2_/FIO_2 _ratio or the oxygenation index when comparing survivors with nonsurvivors (Table 3).

**Table 3:** Comparison of clinical and ventilator data by survival status

Parameter	Survivors (n = 29)	Nonsurvivors (n = 21)	*P *value^a^
Demographics			
Age (years) (mean ± standard deviation)	55 ± 18	56 ± 14	0.76
Gender (% male)	55	57	0.89
Race (% Caucasian)	59	67	0.44
Sepsis as acute lung injury risk (%)	28	57	0.04
Clinical variables (mean ± standard deviation)			
Simplified Acute Physiology Score II	42 ± 13	52 ± 13	0.02
Acute Physiology and Chronic Health Evaluation II	21 ± 6	24 ± 6	0.14
Lung injury score	2.9 ± 0.5	2.7 ± 0.6	0.32
Ventilator variables			
Plateau pressure (cmH_2_O)	26 ± 8	24 ± 5	0.48
Quasistatic respiratory compliance (ml/cmH_2_O)	32 ± 10	30 ± 11	0.60
PaO_2_/FiO_2 _ratio	150 ± 65	162 ± 82	0.54
Oxygenation index	14 ± 10	12 ± 8	0.50

^a^Comparisons were made using chi-square analysis for dichotomous predictor variables and using an unpaired *t *test for continuous predictor variables.

### Biologic markers

**Figure 1:**
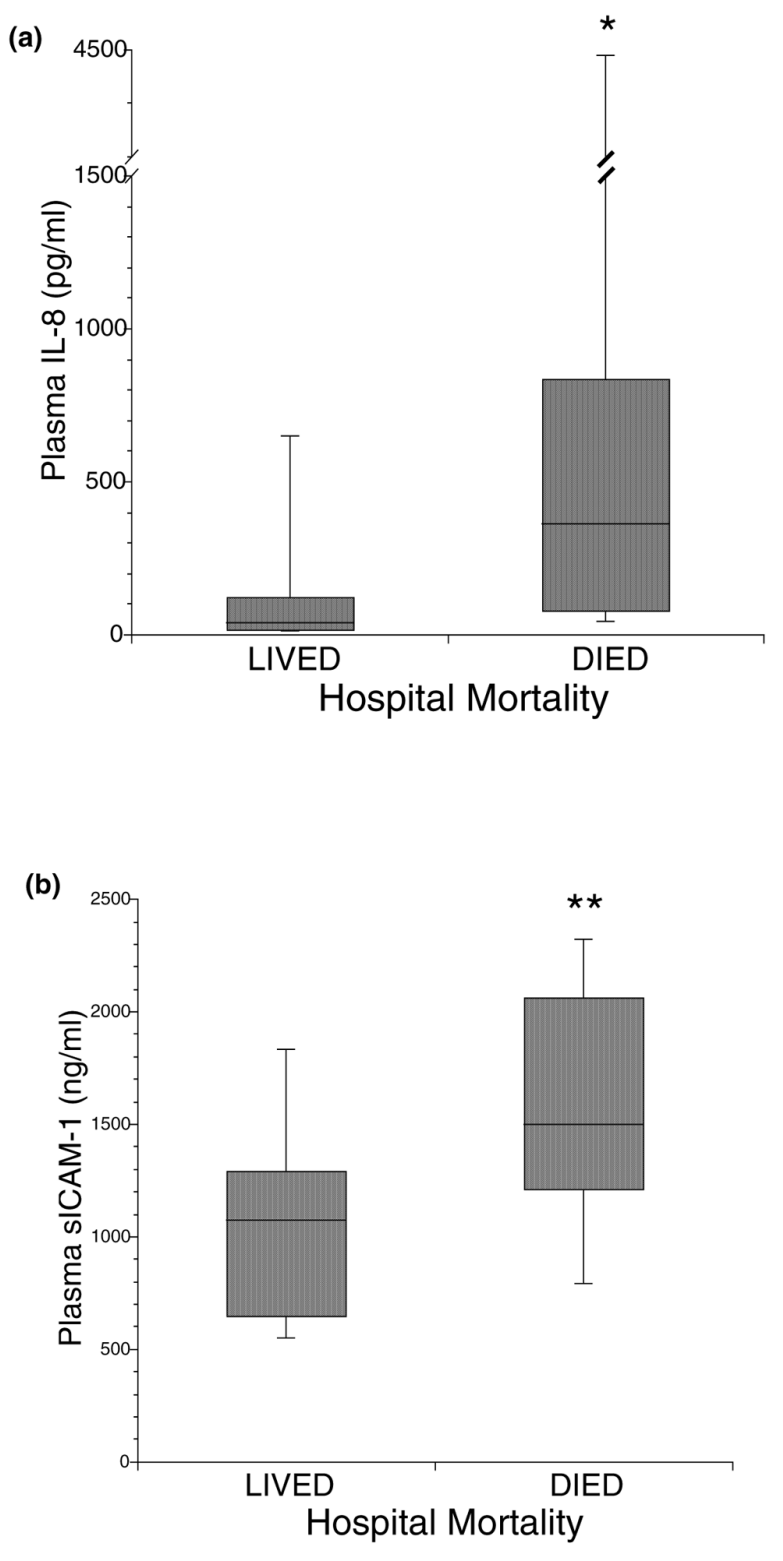
Biomarkers of inflammation in acute lung injury and acute respiratory distress syndrome. Comparison of plasma levels of biomarkers of inflammation in 50 patients with acute lung injury and acute respiratory distress syndrome ventilated with low-tidal-volume ventilation. Plasma levels of **(a) **IL-8 and **(b) **soluble intercellular adhesion molecule 1 (sICAM-1) were significantly higher in nonsurvivors than in survivors. Data shown as boxplots: horizontal line, median; box, 25th to 75th percentiles; error bars, 10th to 90th percentiles. *P = 0.002 and **P = 0.006 compared with survivors, Mann–Whitney U test.

**Figure 2:**
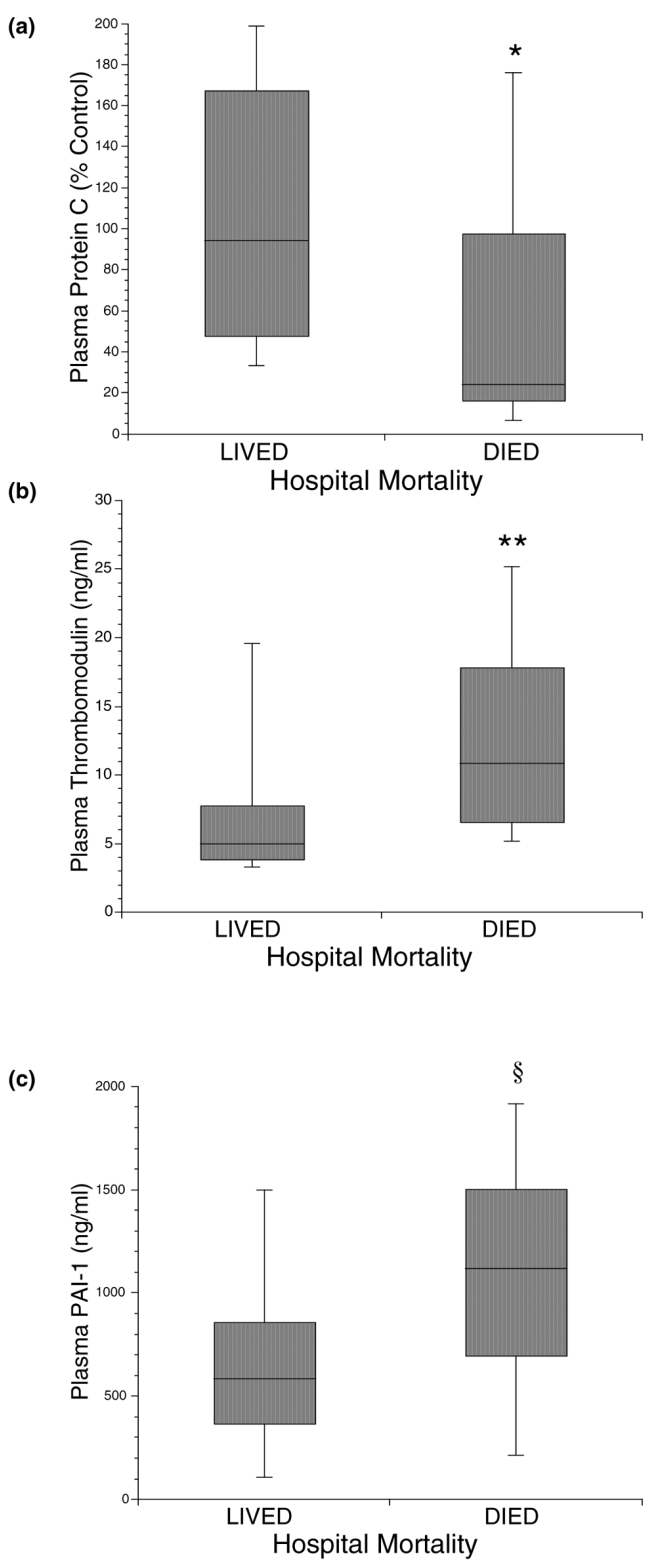
Biomarkers of disordered coagulation and fibrinolysis in acute lung injury and acute respiratory distress syndrome. Comparison of plasma levels of biomarkers of disordered coagulation and fibrinolysis in 50 patients with acute lung injury and acute respiratory distress syndrome ventilated with low-tidal-volume ventilation. **(a) **Plasma levels of protein C were significantly lower in nonsurvivors compared with survivors. **(b) **Plasma levels of thrombomodulin were significantly higher in nonsurvivors compared with survivors. **(c) **Plasma levels of plasminogen activator inhibitor 1 (PAI-1) were significantly higher in nonsurvivors compared with survivors. Data shown as boxplots: horizontal line, median; box, 25th to 75th percentiles; error bars, 10th to 90th percentiles. *P = 0.0003, **P = 0.005 and ^§^*P *= 0.01 compared with survivors, Mann–Whitney U test.

Biomarker levels in survivors versus nonsurvivors are summarized in Figures 1 and 2. All inflammatory biomarkers were elevated in nonsurvivors compared with survivors. The elevations were statistically significant, however, only in the cases of IL-8 and ICAM-1 (*P *= 0.002, *P *= 0.006 respectively; Figure 1). Protein C levels were lower in patients that did not survive as compared with patients that survived (*P *= 0.0003), a finding that indicates greater consumption of this coagulation factor in the group of patients that died (Figure 2a). More severe impairment in coagulation in nonsurvivors was confirmed by evaluation of thrombomodulin levels in this cohort of patients. Higher thrombomodulin levels were demonstrated in patients who did not survive compared with survivors (*P *= 0.005) (Figure 2b). The PAI-1 levels were significantly higher in patients who died compared with those in patients who survived (*P *= 0.01) (Figure 2c).

### Multivariate analysis of clinical and biomarker results

A multivariate logistic regression analysis was performed to evaluate multiple potential contributors to mortality in this patient population. The predictor variables for this analysis included clinical and demographic variables as well as biomarker results. The demographic variables of age, gender and SAPS II score were chosen because of their demonstrated predictive value for outcomes in ALI [15,16]. The biologic markers that were significantly different between groups on bivariate analysis were also included. The final model therefore included age, gender, SAPS II score, IL-8, ICAM-1, thrombomodulin, protein C and PAI-1. Biomarker data were logarithmically transformed prior to inclusion in the multivariate model, given the abnormal distribution.

Elevations in IL-8 and ICAM-1 were independently predictive of increased mortality in patients with ALI, even when considering age, gender, SAPS II score and other biologic marker results (Table 4). Similarly, lower levels of protein C showed a strong trend toward predicting worse clinical outcomes, independent of other predictor variables (Table 4). Logistic regression analysis showed that, for each increase in the natural log of IL-8, the risk of death doubled with an odds ratio of 2.0 (95% confidence interval = 1.1 to 4.0, *P *= 0.04). The risk of death was even higher for ICAM-1. For each natural log increase in the ICAM-1 level, the risk of death increased nearly sixfold (odds ratio = 5.9, 95% confidence interval = 1.1 to 30, *P *= 0.04). Finally, a strong trend was observed for protein C levels, with lower levels associated with worse clinical outcomes. The odds ratio for death decreased by one-half for each natural log increase in the protein C levels (odds ratio = 0.5, 95% confidence interval = 0.2 to 1.0, *P *= 0.06).

**Table 4:** Multivariate logistic regression for clinical and biologic predictors of mortality^a^

Predictor variable	Odds ratio for mortality (per natural log increase in biomarker level)	95% confidence interval	*P *value
IL-8	2.0	1.1 to 4.0	0.04
Intercellular adhesion molecule 1	5.8	1.1 to 30	0.04
Protein C	0.5	0.2 to 1.0	0.06

^a^Multivariate model included age, gender, Simplified Acute Physiology Score II score, log IL-8, log intercellular adhesion molecule 1, log thrombomodulin, log protein C and log plasminogen activator inhibitor 1 to predict the outcome of mortality. Predictor variables not presented in the table showed *P *> 0.3.

To confirm these findings and to evaluate the role of sepsis as a condition predisposing to ALI/ARDS, we carried out a stepwise backward logistic regression for mortality. The analysis included age, gender, SAPS II score, presence or absence of sepsis and each of the biomarkers that showed significant differences between groups in bivariate analyses: IL-8, ICAM-1, protein C, PAI-1 and thrombomodulin. As above, we logarithmically transformed the biomarker variables to create a more normalized distribution. To perform this analysis, we determined *P *values for all variables in the model and then sequentially eliminated the variable with the highest *P *value, as long as the *P *value was >0.20, until the p values for all remaining variables in the model were *P *≤ 0.20. Despite including sepsis in the model, at the end of our analysis the only three variables that remained were log IL-8, log protein, C and log ICAM-1. Using this model, log IL-8 had an odds ratio of 1.6 (95% confidence interval = 1.0 to 2.5, *P *= 0.03), log ICAM-1 had an odds ratio of 2.8 (95% confidence interval = 0.9 to 9.3, *P *= 0.09) and, finally, log protein C had an odds ratio of 0.5 (95% confidence interval = 0.3 to 1.1, *P *= 0.08).

## Discussion

ALI is a complex illness with derangement in multiple metabolic pathways, including inflammation, coagulation and fibrinolysis. Abnormalities of these pathways have been shown in prior evaluations of patients with ALI/ARDS, with the greatest abnormalities presenting in nonsurvivors. These results were obtained, however, before the use of lower tidal volumes and limitations in plateau pressures had been convincingly demonstrated to decrease mortality in clinical ALI/ARDS [12]. Injurious high tidal volumes alone can cause derangements in coagulation and fibrinolysis, and can trigger an inflammatory response [17,18]. To assess abnormalities in inflammation, coagulation and fibrinolysis independent of injurious ventilation, we studied patients at two hospitals that routinely use a low-tidal-volume plateau-pressure-limited ventilatory strategy in patients with ALI/ARDS.

To our knowledge, this is the first study to demonstrate abnormalities in markers of inflammation and impaired coagulation and fibrinolysis remain predictive of increased mortality despite implementation of lung-protective ventilation. Moreover, elevations in IL-8 and ICAM-1 were predictive of increased mortality independent of important clinical predictors and other biomarker abnormalities.

Our findings are consistent with earlier studies of biomarkers in the era prior to routine use of lung-protective mechanical ventilation. ICAM-1 is an adhesion molecule that facilitates trafficking of neutrophils to the lung and is upregulated on the lung endothelial surface during ALI/ARDS [2]. In patients with ALI/ARDS, higher levels of soluble ICAM-1 in the pulmonary edema fluid were associated with an increased length of mechanical ventilation [3]. Higher plasma ICAM-1 levels were also associated with mortality in a prospective study of children with ALI/ARDS [4]. In patients with ALI/ARDS enrolled in a multicenter study of a protective ventilatory strategy, higher baseline levels of IL-6 and IL-8 were associated with increased mortality [5].

Low levels of protein C showed a strong trend for being independently predictive of worse outcome in ALI/ARDS. These findings were confirmed in a rigorous stepwise backward logistic regression model that included sepsis as a covariate. This result is also consistent with prior work in ALI/ARDS. In a small prospective cohort of patients with ALI/ARDS, lower levels of protein C in pulmonary edema fluid were associated with increased mortality [7], as were lower plasma levels in a larger multicenter cohort [10] regardless of the presence or absence of sepsis. We therefore believe that protein C is associated with outcomes in ALI/ARDS and is not simply reflective of higher numbers of patients with sepsis in the nonsurvivor group. This finding suggests that protein C administration in patients with ALI/ARDS may have some benefit; however, a recent phase II, randomized controlled trial of activated protein C administration in patients with ALI/ARDS was stopped early because of lack of efficacy in the treatment group over placebo (Michael Matthay, unpublished data). Further work to understand the role of protein C in ALI/ARDS is therefore indicated.

PAI-1 levels were significantly higher in nonsurvivors than survivors on bivariate analysis. This confirms previous work in ALI examining PAI-1 levels in the era prior to routine use of low-tidal-volume ventilation. Prior work has demonstrated decreased urokinase activity in the air spaces of patients with ALI/ARDS, and this decrease is explained by elevations in levels of PAI-1 [8]. In a small single-center study, PAI-1 levels in plasma and pulmonary edema samples from patients with ALI/ARDS were associated with higher mortality rates [9]; this finding was confirmed in a larger multicenter study [10]. In the current study, the PAI-1 levels did not remain independently predictive on multivariate analyses. This finding may reflect the relatively small sample size.

Lung-protective ventilation, although clearly demonstrated to improve survival in ALI/ARDS [12], has not been routinely adopted as standard of care [19,20]. To confirm that patients in our cohort were ventilated with a low-tidal-volume protocol, we compared the ventilator settings for patients in our study with data from the original ARDS Network trial of lower-tidal-volume ventilation. The ventilator parameters were nearly identical for patients in our study compared with patients in the original trial, with a mean tidal volume of 6.6 ml/kg predicted body weight and a mean inspiratory plateau pressure of 25 cmH_2_O. One additional similarity between the current study population and the ARDS Network trial population was that the PaO_2_/FiO_2 _ratio was lower (worse) in both cases in the groups that survived, although the result was not statistically significant. These data confirm that the PaO_2_/FiO_2 _ratio is not a good surrogate for outcomes in ALI.

Bivariate analysis of each of biomarker demonstrated that higher levels of IL-8, ICAM-1, thrombomodulin and PAI-1 and lower levels of protein C were significantly associated with increased mortality. The higher levels of IL-8 and ICAM-1 suggest there is greater upregulation of the acute inflammatory process in patients with ALI/ARDS who did not survive their illness. Similarly, lower protein C and higher thrombomodulin levels indicate greater activation of coagulation pathways in patients who died. Finally, the significantly higher level of PAI-1 in patients who died indicates greater impairment of fibrinolysis in these patients. Given that these patients were maintained on lung-protective ventilation, ventilator-induced lung injury is not a probable explanation for the abnormalities of inflammation and coagulation.

Parsons and colleagues [5] demonstrated in ARDS Network patients that low-tidal-volume ventilation was associated with lower levels of plasma IL-6 and IL-8 levels by day 3 of the study compared with patients maintained on 12 ml/kg. Protein C levels were also normalized to a greater extent in the low-tidal-volume group [10]. A remaining possibility, however, is that even a lung-protective ventilator strategy is injurious in the acutely injured lung. In a rat model of acid-induced lung injury, Frank and colleagues showed that lung endothelial and epithelial injury were minimized by a reduction in tidal volume to 3 ml/kg compared with 6 or 12 ml/kg [17], suggesting that even a 6 ml/kg tidal volume might be injurious in some patients.

There are some limitations to our study. First, we studied a relatively small cohort of patients from two hospitals. For this reason, the study may have been underpowered to show a significant association between lower levels of protein C and adverse clinical outcomes in the multivariable analyses. Second, data were not collected on the ventilatory strategy employed prior to enrolment in the study. Patients were enrolled within 48 hours of meeting diagnostic criteria for ALI/ARDS so there was a maximum of 2 days in which patients may have received injurious ventilation. We therefore cannot rule out injurious ventilation prior to enrolment in the study possibly contributing to the findings of the study. Third, the biomarkers we studied were logarithmically transformed to enable statistical analysis. In practice, this means that a large increase in a biomarker such as IL-8 level is associated with a somewhat smaller increased risk of death. The results from the present study are therefore more likely to be useful in understanding the pathogenesis and ongoing injury during ALI/ARDS than as a diagnostic test for individual patients with ALI/ARDS.

In summary, the association of the biologic markers with adverse clinical outcomes does not confirm causality, but rather suggests important *in vivo *pathways for further study. In addition to clinical utility for prognostication and stratification of patients for enrollment in clinical trials, the clinical measurement of biomarkers may help to elucidate mechanisms of human disease that may have value in designing new therapies for ALI/ARDS.

## Conclusion

Plasma biomarkers that are related to inflammation and enhanced neutrophil recruitment to the lung are independently associated with increased mortality in patients with ALI. The borderline significant association of lower protein C levels with nonsurvivors continues to support the role for disordered coagulation in ALI/ARDS. These associations exist despite consistent use of lung-protective ventilation and persist even when controlling for clinical factors that also impact upon outcomes. The two biomarkers with an independent association with mortality, IL-8 and ICAM-1, should be studied further for their potential value in stratifying patients in clinical trials.

## Key messages

• In a group of ARDS patients treated with strict low-tidal-volume ventilation, plasma biomarkers that are related to inflammation (IL-8) and to enhanced neutrophil recruitment to the lung (ICAM-1) are independently associated with increased mortality in patients with ALI.

• The trend towards independent association of lower protein C levels with nonsurvivors supports the role for disordered coagulation in ALI/ARDS.

• Despite lung-protective ventilation, abnormalities in plasma levels of markers of inflammation, coagulation and fibrinolysis predict mortality in ALI/ARDS patients, indicating more severe activation of these biologic pathways in nonsurvivors.

## Abbreviations

ALI = acute lung injury; ARDS = acute respiratory distress syndrome; ELISA = enzyme-linked immunosorbent assay; ICAM-1 = intercellular adhesion molecule 1; IL = interleukin; PAI-1 = plasminogen activator inhibitor 1; PaO_2_/FiO_2 _= ratio of arterial to inspired oxygen; SAPS II = Simplified Acute Physiology Score II.

## Competing interests

The authors declare that they have no competing interests.

## Authors' contributions

DM conceived the study, enrolled the patients, collected the samples, interpreted the data and drafted the manuscript. HJZ assisted with the biostatistical analysis. NW carried out the immunoassays. MAM and LBW conceived the study, participated in its design and coordination and helped to draft the manuscript. All authors read and approved the final manuscript.
